# The Role of Bacteria and Pattern Recognition Receptors in GvHD

**DOI:** 10.4061/2010/814326

**Published:** 2010-10-31

**Authors:** E. Holler, K. Landfried, J. Meier, M. Hausmann, G. Rogler

**Affiliations:** ^1^Department of Haematology/Oncology, University of Regensburg, 93042 Regensburg, Germany; ^2^Department of Cranio-Maxillofacial Surgery, University of Regensburg, 93042 Regensburg, Germany; ^3^Division of Gastroenterology and Hepatology, University of Zurich, 8091 Zurich, Switzerland

## Abstract

Graft-versus-Host Disease (GvHD) is the most serious complication of allogeneic stem cell transplantation (SCT) and results from an activation of donor lymphocytes by recipient antigen-presenting cells (APCs). For a long time, it has been postulated that the intestinal microflora and endotoxin exert a crucial step in this APC activation, as there is early and severe gastrointestinal damage induced by pretransplant conditioning. With the detailed description of pathogen-associated molecular patterns and pathogen recognition receptors single nucleotide polymorphisms of TLRs and especially NOD2 have been identified as potential risk factors of GvHD and transplant related complications thus further supporting the crucial role of innate immunity in SCT, related complications. Gastrointestinal decontamination and neutralization of endotoxin have been used to interfere with this early axis of activation with some success but more specific approaches of modulation of innate immunity are needed for further improvement of clinical outcome.

## 1. Introduction

Graft-versus-Host Disease (GvHD) in its acute and chronic form is the major cause of mortality and morbidity following both, experimental and clinical allogeneic stem cell transplantation (SCT). Donor T-cells activated by major or minor histocompatibility antigens on host antigen presenting cells (APCs) are the essential players in the pathophysiology of GvHD [1], and T-cell depletion of the graft is able to abrogate both, GvHD and the beneficial graft-versus-leukemia (GvL) effect. However, it has been well known since the fundamental experiments of van Bekkum that activation of innate immunity by the gastrointestinal microflora is a crucial and initiating step in induction of alloreactions. Mice grown under germ-free conditions and receiving bone marrow as the only stem cell source (containing a limited number of T-cells) failed to develop acute GvHD whereas, mice grown under conventional conditions or reconventionalized early after transplantation died from acute GvHD. If spleen cells containing a high number of T-cells were added, germ-free conditions could not prevent but still delayed the onset of GvHD [2]. Since this first observation substantial progress has been made in understanding the exact pathways how bacteria and their ligands interact with specific pattern recognition receptors (PRRs), thus activating and modulating APCs and targets of GvHD. In the present paper we summarize current evidence on the impact of microbia and microbial patterns on pathophysiology of GvHD and clinical outcome following allogeneic SCT.

## 2. Indirect Evidence: Gastrointestinal Damage and GvHD

Major target organs of acute GvHD are the skin, the liver, especially bile duct epithelia, the gastrointestinal tract and still controversially discussed, the lung. In chronic GvHD, oral manifestations and again, the pulmonary involvement of bronchiolitis obliterans (BOs) are frequent. A common denominator of these organs is that they present epithelial surfaces with strong immunological interactions between commensal and pathogenic bacteria, epithelial barrier and defence mechanisms and the immune system which are usually in a perfect balance to maintain a status of immunological tolerance [3]. 

These epithelial defence mechanisms are heavily disturbed by epithelial damage through pretransplant conditioning which is the first step in the SCT procedure and includes high-dose cytotoxic therapy or total body irradiation (TBI). Although conditioning aims to eliminate the recipient's leukemia and achieve myeloablation, in both, experimental and clinical SCT, it has become clear that increasing the dose of TBI from 900 to 1300 cGy in mice and from 12 to 15,5 Gy clinically was associated with increased epithelial damage, more severe GvHD and inferior outcome [4–6]. Similarly, an increased area under the curve (AUC) following exposure to intravenous busulfan was also associated with increased gastrointestinal toxicity and acute GvHD [7].

In 2 clinical trials, gastrointestinal toxicity was directly assessed by either monitoring diarrhea during the aplastic phase [8] or direct analysis of intestinal permeability [9] and could be correlated with severity of subsequent acute GvHD. In line with this, prophylactic treatment of mice with the rhu Keratinocyte Growth Factor (KGF) protected from apoptosis of intestinal epithelial cells, LPS mediated TNF release, and finally lethal GvHD [10] while maintaining GvL effects [11]. However, in a randomized clinical trial KGF reduced severity of mucositis in patients receiving TBI but did not affect GvHD and outcome as expected from murine data [12].

## 3. Indirect Evidence: The Role of SNPs of Innate Immunity

In murine GvHD, endotoxin has been identified as a major mediator of inflammation involved in initiation of intestinal and systemic GvHD. Genetic susceptibility as well as direct antagonisms against endotoxin has a major impact on the occurrence and severity of experimental GvHD [5, 13] and an LPS-TNF*α* axis has been postulated as a major mechanism of acute GvHD. In humans, TLR4 is considered to represent the classical endotoxin receptor. With the characterization of the human genome, single nucleotide polymorphisms (SNPs) for many genes have been described which translate into altered functions of these genes. In the setting of allogeneic SCT, TLR4 SNPs have been assessed by 2 groups which however, reported opposing effects, either protection or enhancement of GvHD in the presence of TLR4 SNPs [14, 15].

**Figure 1:**
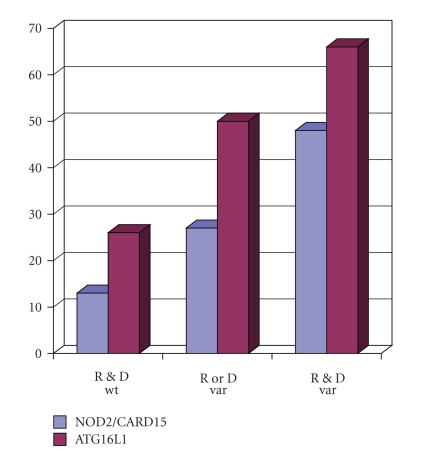
Treatment-related mortality and SNPs of innate immunity. SNPs 8,12 and 13 of NOD2 (*n* = 358) and the T300A. SNP of ATG16L1 (*n* = 127) were assessed by PCR in patients receiving allogeneic SCT and their respective HLA-identical sibling donors. Cumulative treatment related mortality as calculated by Kaplan-Meier method is shown in relation to absence of any SNPs (wt, wild-type) or presence of SNPs (variant, var) in recipients (R) and/or donors (D). Both associations were significant by log rank tests (*P* = .003 for NOD2, *P* = .03 for ATG16L1).

Beyond TLR4, there is a large set of further TLRs recognizing other microbial patterns including further bacterial and viral ligands. SNPs have been described for most of these TLRs, and recently, presence of the homozygous TLR9 variant in the patient has been associated with improved survival and a reduced relapse rate following allogeneic SCT [16]. In addition to TLRs, the NOD-like receptors are a family of highly conserved intracytoplasmatic receptors involved in activation of an important inflammatory cascade, the inflammasome, which finally results in activation of NF-*κ*B and/or activation and cleavage of IL-1*β* [17]. Based on pathophysiological similarities between inflammatory bowel disease and intestinal GvHD, we speculated that SNPs involved in the pathogenesis of Crohn's disease might be also of relevance in GvHD and outcome following allogeneic SCT [18]. We and others have therefore tested SNPs of NOD2, a receptor sensing muramyl-dipeptide derived from Gram-positive and Gram-negative bacteria, as well as SNPs of other innate immunity molecules such as the autophagy-related gene 1 (ATG16L1) involved in autophagy of bacteria [18], in large cohorts of patients receiving HLA-identical sibling SCT and their donors. Presence of any NOD2 SNPs or the ATG16L1 variants in either the recipient or the donor increased the risk of GvHD and subsequent treatment related mortality, and this effect was further increased if variants were present in both, donor and recipient (Figure 1). 

Since our first description of association of NOD2 variants with GvHD and mortality [19] several further groups addressed the role of this important receptor. In HLA-identical sibling transplants, most of the studies were confirmatory [20–24]; in unrelated donor transplantation, the association either was absent [25], present only for SNP13 [26] or even contradictory as one group reported association with less GvL and more relapse [27–29] (Table 1). This may be explained by the fact that in unrelated donor SCT HLA-differences are much more likely to occur and dominant against the SNPs of innate immunity or by the more intense immunosuppression which usually includes in vivo T-cell depletion with monoclonal or polyclonal sera. In addition, our European cohort analysis nicely demonstrated that differences in transplant specific strategies, especially with regard to antibacterial decontamination, had a strong impact on the prognostic significance of NOD2. Thus, immunoregulatory SNPs may be specifically sensitive to interaction with transplant or center-specific strategies and it may be therefore difficult to establish these SNPs as risk factors allowing exact prediction of complications.

However, a further and even more important aspect of these observations is the implication of these molecules in pathophysiology. Our observation of an association of bronchiolitis obliterans syndrome with NOD2 SNPs suggest that this interference of SNPs with epithelial defence mechanisms applies to all epithelial tissues [30], and it will be of major interest to learn more about antibacterial peptides released like defensins released under the control of NOD2 [31]. Recently, NOD2^−/−^ mice and bone marrow chimeras were used as recipients in experimental BMT models. These data confirmed an accelerated mortality from GvHD if hematopoietic cells were NOD2 deficient, and O' Penack and his group identified the antigen-presenting cell (APC) as the target of NOD2 deficiency [32]. APCs from NOD2^−/−^ mice induced a much stronger alloreaction as compared to wild-type mice pointing to a deficiency of immunoregulatory APC molecules. Recently, our group addressed the immunohistopathology of skin and gut GvHD in relation to absence and presence of NOD2 SNPs: In both tissues, skin and gut, recipient NOD2 SNPs had no impact on the extent of apoptosis as the hallmark of GvHD nor on the CD8 and macrophage infiltrate in the biopsies. However, we observed a uniform reduction of CD4 cells in the presence of NOD2 SNPs suggesting that GvHD especially in these patients is characterized by a loss of protective CD4 population including a loss of regulatory T-cells [33]. NOD2 has been shown to be involved in release of chemokines attracting T-helper cells as well as in recruitment of TH17 cells, and ongoing studies try to address these questions. Thus, a dysregulated APC function is the most likely explanation for this loss of CD4 cells as it has been observed in mice.

## 4. Direct Evidence: The Role of Bacteria and Bacterial Ligands

van Bekkum's work elegantly elaborated the role of the intestinal microflora and is up to now the basis for the gnotobiotic approach to modulate GvHD. In his early experiments he did not only show protection from GvHD induced by bone marrow transplantation in gnotobiotic mice. He also reported that subcutaneous fetal gut implants revealed attenuated histopathological GvHD if the carrier mice were germ-free prior to allogeneic transplantation indicating a systemic effect of decontamination [34]. Similar observations pointing to a systemic modulation [35] were reported by Lampert et al. [35], as they reported attenuation of both gut and skin GvHD after oral decontamination in mice. In the same year, Veenendaal et al. published strain-dependent effects of decontamination, as C3H/He recipients were protected from delayed type GvHD by decontamination whereas C57Bl/6 were not [36]. Later on, a debate started whether selective decontamination versus decontamination including anaerobic bacteria conferred greater protection from GvHD. In both, experimental BMT [37] and in clinical trials published by the Essen group [38, 39] suppression of the anearobic flora seemed to have a stronger effect than suppression of enterobacteriaceae alone. 

Further evidence for a role of the intestinal flora can be derived from attempts to neutralize endotoxin. In 1987, Cohen reported protection of mice by passive immunization with anti-*E.coli* sera, and an increased antibody titer against a certain *E. coli* strain in patients was associated with a lower incidence of severe GvHD [40, 41]. In the 90s, IgM enriched immunoglobulin preparations were thought to reduce GvHD due to their potent antiendotoxin effects [42].

Based on positive reports in inflammatory bowel disease, our group tested the use of probiotic bacteria (*Lactobacillus rhamnosus*) in an experimental BMT model. Indeed, feeding of lactobacilli reduced severity of experimental GvHD and improved survival. The systemic effect of probiotic bacteria could be demonstrated by a reduction of splenic donor T-cell proliferation further demonstrating that activation of innate immunity sets the state for subsequent adaptive alloreactions [43].

Besides endotoxin modulation, only rare reports have addressed the role of other bacterial and TLR ligands. In an elegant study, Chakraverty et al. proved the checkpoint function for innate immunity by applying a TLR7 activator locally to the skin before inducing GvHD by donor lymphocyte infusion in mixed murine chimeras. Whereas there were almost no cellular infiltrates and signs of GvHD in untreated control skin, massive T-cell infiltrates and histopathological damage was observed in the TLR7ligand pretreated skin [44]. Similar processes of activation should occur in the intestinal tract and in other epithelial target tissues of GvHD after TLR4 and TLR2 binding of endotoxins. In line with the checkpoint function of innate immunity, binding of CPG-oligodeoxynucleotides to host antigen-presenting cells accelerated GvHD in a recent murine study [45], whereas murine TLR9^−/−^ recipients were protected from GvHD [46]. Interestingly, other TLR-ligands seem to induce opposing and even silencing effects. Pretreatment of mice with the TLR5 ligand flagellin reduced severity of GvHD indicating that TLR5 may be more involved in dampening activation of antigen presenting cells (Gerwitz, ASBMT 2010, abstract).

## 5. Perspective: Modulation of PRRs to Avoid GvHD While Preserving GvL Effects

Since many years, attempts to reduce GvHD frequently also affected the major therapeutic principle of allogeneic SCT, the graft-versus-leukemia effect. This was most obvious for direct T-cell depletion [47] but can also be observed for classical immunosuppressants like cyclosporin. Especially in acute leukemias, occurrence of mild acute GvHD grade I-II and chronic GvHD confers the best antileukemic effect. In our studies on the role of NOD2 SNPs, however, we observed a strong impact of recipient and combined donor/recipient SNPs on GvHD, and GvHD-related mortality, however, there was no difference in relapse rates between the different groups. Patients with wild-type NOD2 had a cumulative incidence of relapse of 41%, patients with either recipient or donor variants of 29% and patients with combined donor and recipient variants 33% [20]. This observation and pathophysiological considerations suggest that modulation of epithelial inflammation in the gut or the bronchial epithelial system thus might reduce GvHD but should not interfere with antileukemic immunity which is located in the central lymph nodes or in the marrow.

## 6. Conclusions

Although our current understanding of the interplay between intestinal microbes, activation of innate immunity, and specific alloreactions explains some of the long standing preclinical and clinical findings such as the potential protective effect of intestinal decontamination, there are still a variety of issues to be solved. Comparable to the situation in IBD, SNPs of innate immunity alone by far do not explain the individual susceptibility for intestinal GvHD or allow even prediction of intestinal GvHD which would be extremely helpful to tailor immunosuppressive prophylaxis and treatment suggesting that intestinal homeostasis is far more complex. In addition, the role of the diversity of the intestinal microbiota and the impact of immunological memory against these antigens in GvHD has not been addressed so far. In addition, pathophysiology has focussed on excess inflammation so far. As suggested by our findings on a reduction of intestinal regulatory T-cells in patients with NOD2 SNPs and GvHD, GvHD may be far more a loss of intestinal immunoregulation, and mechanisms of immunoregulation need to be investigated in detail. Recent data indicate that the balance of regulatory to TH17 cells is strongly regulated by the enzyme Indolamine-2,3 dioxygenase (IDO) in intestinal antigen-presenting cells. Experimental data suggest a strong impact of this enzyme in GvHD pathophysiology [48], and ongoing studies address this new player in the clinical setting. In the long term, these findings should help to substitute nonspecific immunosuppression for treatment of intestinal GvHD by strategies aiming at reconstitution of immunoregulation.

## Figures and Tables

**Table 1:** Summary of published studies on NOD2 SNPs and outcome following SCT.

	Type of SCT	Association	Comment	Refereces
Holler 2004	Related	GvHD, TRM	Single centre	[17]
Holler 2006	Related	GvHD, TRM, OS	Multicentre; Impact of decontamination	[18]
Granell 2006	Related	TRM, pulmonary compl.	CD34 selected grafts	[19]
Sairafi 2008	Related	No association	Low frequency of NOD2 variants	[22]
Hanssen 2008	Related	Weak with GvHD		[20]
Van Velden 2009	Related	Strong with GvHD	Partially T depleted Grafts	[21]
Hildebrandt 2009	Related and Unrelated	Bronchiolitis obliterans		[28]
Mayor 2009	Unrelated	Strong with relapse, not with GvHD	Majority received T-cell depletion With MabCampath	[25]
Holler 2009	Unrelated	Only SNP13 with TRM		[24]
Ngyen 2010	Unrelated	No		[23]

## References

[1] Ferrara JL, Levine JE, Reddy P, Holler E (2009). Graft-versus-host disease. *Lancet*.

[2] van Bekkum DW, Roodenburg J, Heidt PJ, van der Waaij D (1974). Mitigation of secondary disease of allogeneic mouse radiation chimeras by modification of the intestinal microflora. *Journal of the National Cancer Institute*.

[3] Wells JM, Loonen LMP, Karczewski JM (2010). The role of innate signaling in the homeostasis of tolerance and immunity in the intestine. *International Journal of Medical Microbiology*.

[4] Hill GR, Ferrara JLM (2000). The primacy of the gastrointestinal tract as a target organ of acute graft-versus-host disease: rationale for the use of cytokine shields in allogeneic bone marrow transplantation. *Blood*.

[5] Cooke KR, Gerbitz A, Crawford JM (2001). LPS antagonism reduces graft-versus-host disease and preserves graft-versus-leukemia activity after experimental bone marrow transplantation. *Journal of Clinical Investigation*.

[6] Clift RA, Buckner CD, Appelbaum FR (1990). Allogeneic marrow transplantation in patients with acute myeloid leukemia in first remission: a randomized trial of two irradiation regimens. *Blood*.

[7] Andersson BS, Thall PF, Madden T (2002). Busulfan systemic exposure relative to regimen-related toxicity and acute graft-versus-host disease: defining a therapeutic window for IV BuCy2 in chronic myelogenous leukemia. *Biology of Blood and Marrow Transplantation*.

[8] Goldberg J, Jacobsohn DA, Zahurak ML, Vogelsang GB (2005). Gastrointestinal toxicity from the preparative regimen is associated with an increased risk of graft-versus-host disease. *Biology of Blood and Marrow Transplantation*.

[9] Johansson J-E, Ekman T (2007). Gut toxicity during hemopoietic stem cell transplantation may predict acute graft-versus-host disease severity in patients. *Digestive Diseases and Sciences*.

[10] Ellison CA, Natuik SA, Fischer JMM (2004). Effect of recombinant human keratinocyte growth factor (rHuKGF) on the immunopathogenesis of intestinal graft-vs.-host disease induced without a preconditioning regimen. *Journal of Clinical Immunology*.

[11] Krijanovski OI, Hill GR, Cooke KR (1999). Keratinocyte growth factor separates graft-versus-leukemia effects from graft-versus-host disease. *Blood*.

[12] Blazar BR, Weisdorf DJ, DeFor T (2006). Phase 1/2 randomized, placebo-control trial of palifermin to prevent graft-versus-host disease (GVHD) after allogeneic hematopoietic stem cell transplantation (HSCT). *Blood*.

[13] Cooke KR, Olkiewicz K, Erickson N, Ferrara JLM (2002). The role of endotoxin and the innate immune response in the pathophysiology of acute graft versus host disease. *Journal of Endotoxin Research*.

[14] Elmaagacli AH, Koldehoff M, Hindahl H (2006). Mutations in innate immune system NOD2/CARD 15 and TLR-4 (Thr399Ile) genes influence the risk for severe acute graft-versus-host disease in patients who underwent an allogeneic transplantation. *Transplantation*.

[15] Lorenz E, Schwartz DA, Martin PJ (2001). Association of TLR4 mutations and the risk for acute GVHD after HLA-matched-sibling hematopoietic stem cell transplantation. *Biology of Blood and Marrow Transplantation*.

[16] Elmaagacli AH, Koldehoff M, Beelen DW (2009). Improved outcome of hematopoietic SCT in patients with homozygous gene variant of Toll-like receptor 9. *Bone Marrow Transplantation*.

[17] Martinon F, Mayor A, Tschopp J (2009). The inflammasomes: guardians of the body. *Annual Review of Immunology*.

[18] Hampe J, Franke A, Rosenstiel P (2007). A genome-wide association scan of nonsynonymous SNPs identifies a susceptibility variant for Crohn disease in ATG16L1. *Nature Genetics*.

[19] Holler E, Rogler G, Herfarth H (2004). Both donor and recipient NOD2/CARD15 mutations associate with transplant-related mortality and GvHD following allogeneic stem cell transplantation. *Blood*.

[20] Holler E, Rogler G, Brenmoehl J (2006). Prognostic significance of NOD2/CARD15 variants in HLA-identical sibling hematopoietic stem cell transplantation: effect on long-term outcome is confirmed in 2 independent cohorts and may be modulated by the type of gastrointestinal decontamination. *Blood*.

[21] Granell M, Urbano-Ispizua A, Aróstegui JI (2006). Effect of NOD2/CARD15 variants in T-cell depleted allogeneic stem cell transplantation. *Haematologica*.

[22] van der Velden WJFM, Blijlevens NMA, Maas FMHM (2009). NOD2 polymorphisms predict severe acute graft-versus-host and treatment-related mortality in T-cell-depleted haematopoietic stem cell transplantation. *Bone Marrow Transplantation*.

[23] Hansen JA, Petersdorf EW, Lin M-T (2008). Genetics of allogeneic hematopoietic cell transplantation. Role of HLA matching, functional variation in immune response genes. *Immunologic Research*.

[24] Sairafi D, Uzunel M, Remberger M, Ringdén O, Mattsson J (2008). No impact of NOD2/CARD15 on outcome after SCT. *Bone Marrow Transplantation*.

[25] Nguyen Y, Al-Lehibi A, Gorbe E (2010). Insufficient evidence for association of NOD2/CARD15 or other inflammatory bowel disease-associated markers on GVHD incidence or other adverse outcomes in T-replete, unrelated donor transplantation. *Blood*.

[26] Holler E, Rogler G, Brenmoehl J (2008). The role of genetic variants of NOD2/CARD15, a receptor of the innate immune system, in GvHD and complications following related and unrelated donor haematopoietic stem cell transplantation. *International Journal of Immunogenetics*.

[27] Mayor NP, Shaw BE, Hughes DA (2007). Single nucleotide polymorphisms in the NOD2/CARD15 gene are associated with an increased risk of relapse and death for patients with acute leukemia after hematopoietic stem-cell transplantation with unrelated donors. *Journal of Clinical Oncology*.

[28] Mayor NP, Shaw BE, Madrigal JA, Marsh SGE (2008). No impact of NOD2/CARD15 on outcome after SCT: a reply. *Bone Marrow Transplantation*.

[29] Holler E, Hahn J, Andreesen R (2008). NOD2/CARD15 polymorphisms in allogeneic stem-cell transplantation from unrelated donors: T depletion matters. *Journal of Clinical Oncology*.

[30] Hildebrandt GC, Granell M, Urbano-Ispizua A (2008). Recipient NOD2/CARD15 variants: a novel independent risk factor for the development of bronchiolitis obliterans after allogeneic stem cell transplantation. *Biology of Blood and Marrow Transplantation*.

[31] Wehkamp J, Koslowski M, Wang G, Stange EF (2008). Barrier dysfunction due to distinct defensin deficiencies in small intestinal and colonic Crohn’s disease. *Mucosal Immunology*.

[32] Penack O, Smith OM, Cunningham-Bussel A (2009). NOD2 regulates hematopoietic cell function during graft-versus-host disease. *Journal of Experimental Medicine*.

[33] Landfried K, Bataille F, Rogler G (2010). Recipient NOD2/CARD15 status affects cellular infiltrates in human intestinal graft-versus-host disease. *Clinical and Experimental Immunology*.

[34] van Bekkum DW, Knaan S (1977). Role of bacterial microflora in development of intestinal lesions from graft versus host reaction. *Journal of the National Cancer Institute*.

[35] Lampert IA, Moore RH, Huby R, Cohen J (1988). Observations on the role of endotoxin in graft-versus-host disease. *Progress in Clinical and Biological Research*.

[36] Veenendaal D, de Boer F, van der Waaij D (1988). Effect of selective decontamination of the digestive tract of donor and recipient on the occurrence of murine delayed-type graft-versus-host disease. *Medical Microbiology and Immunology*.

[37] Heidt PJ, Vossen JM (1992). Experimental and clinical gnotobiotics: influence of the microflora on graft-versus-host disease after allogeneic bone marrow transplantation. *Journal of Medicine*.

[38] Beelen DW, Haralambie E, Brandt H (1992). Evidence that sustained growth suppression of intestinal anaerobic bacteria reduces the risk of acute graft-versus-host disease after sibling marrow transplantation. *Blood*.

[39] Beelen DW, Elmaagacli A, Müller K-D, Hirche H, Schaefer UW (1999). Influence of intestinal bacterial decontamination using metronidazole and ciprofloxacin or ciprofloxacin alone on the development of acute graft- versus-host disease after marrow transplantation in patients with hematologic malignancies: final results and long-term follow-up of an open-label prospective randomized trial. *Blood*.

[40] Cohen J, Moore RH, Al Hashimi S, Jones L, Apperley JF, Aber VR (1987). Antibody titres to a rough-mutant strain of Escherichia coli in patients undergoing allogeneic bone-marrow transplantation. Evidence of a protective effect against graft-versus-host disease. *Lancet*.

[41] Moore RH, Lampert IA, Chia Y, Aber VR, Cohen J (1987). Effect of immunization with Escherichia coli J5 on graft-versus-host disease induced by minor histocompatibility antigens in mice. *Transplantation*.

[42] Klingemann H-G, Barnett MJ, Reece DE, Shepherd JD, Phillips GL (1990). Use of an immunoglobulin preparation enriched for IgM (Pentaglobin) for the treatment of acute graft-versus-host disease. *Bone Marrow Transplantation*.

[43] Gerbitz A, Schultz M, Wilke A (2004). Probiotic effects on experimental graft-versus-host disease: let them eat yogurt. *Blood*.

[44] Chakraverty R, Côté D, Buchli J (2006). An inflammatory checkpoint regulates recruitment of graft-versus-host reactive T cells to peripheral tissues. *Journal of Experimental Medicine*.

[45] Taylor PA, Ehrhardt MJ, Lees CJ (2008). TLR agonists regulate alloresponses and uncover a critical role for donor APCs in allogeneic bone marrow rejection. *Blood*.

[46] Calcaterra C, Sfondrini L, Rossini A (2008). Critical role of TLR9 in acute graft-versus-host disease. *Journal of Immunology*.

[47] Horowitz MM, Gale RP, Sondel PM (1990). Graft-versus-leukemia reactions after bone marrow transplantation. *Blood*.

[48] Jasperson LK, Bucher C, Panoskaltsis-Mortari A (2008). Indoleamine 2,3-dioxygenase is a critical regulator of acute graft-versus-host disease lethality. *Blood*.

